# Identification of response-modulated genetic interactions by sensitivity-based epistatic analysis

**DOI:** 10.1186/1471-2164-11-493

**Published:** 2010-09-10

**Authors:** Cory Batenchuk, Lioudmila Tepliakova, Mads Kærn

**Affiliations:** 1 Ottawa Institute of Systems Biology, University of Ottawa, 451 Smyth Road, Ottawa, Ontario, K1H 8M5, Canada; 2 Department of Cellular and Molecular Medicine, University of Ottawa, 451 Smyth Road, Ottawa, Ontario, K1H 8M5, Canada; 3 Department of Physics, University of Ottawa, 150 Louis Pasteur, Ottawa, Ontario, K1N 6N5, Canada

## Abstract

**Background:**

High-throughput genomics has enabled the global mapping of genetic interactions based on the phenotypic impact of combinatorial genetic perturbations. An important next step is to understand how these networks are dynamically remodelled in response to environmental stimuli. Here, we report on the development and testing of a method to identify such interactions. The method was developed from first principles by treating the impact on cellular growth of environmental perturbations equivalently to that of gene deletions. This allowed us to establish a novel neutrality function marking the absence of epistasis in terms of sensitivity phenotypes rather than fitness. We tested the method by identifying fitness- and sensitivity-based interactions involved in the response to drug-induced DNA-damage of budding yeast *Saccharomyces cerevisiae *using two mutant libraries - one containing transcription factor deletions, and the other containing deletions of DNA repair genes.

**Results:**

Within the library of transcription factor deletion mutants, we observe significant differences in the sets of genetic interactions identified by the fitness- and sensitivity-based approaches. Notably, among the most likely interactions, only ~50% were identified by both methods. While interactions identified solely by the sensitivity-based approach are modulated in response to drug-induced DNA damage, those identified solely by the fitness-based method remained invariant to the treatment. Comparison of the identified interactions to transcriptional profiles and protein-DNA interaction data indicate that the sensitivity-based method improves the identification of interactions involved in the DNA damage response. Additionally, for the library containing DNA repair mutants, we observe that the sensitivity-based method improves the grouping of functionally related genes, as well as the identification of protein complexes, involved in DNA repair.

**Conclusion:**

Our results show that the identification of response-modulated genetic interactions can be improved by incorporating the effect of a changing environment directly into the neutrality function marking the absence of epistasis. We expect that this extension of conventional epistatic analysis will facilitate the development of dynamic models of gene networks from quantitative measurements of genetic interactions. While the method was developed for growth phenotype, it should apply equally well for other phenotypes, including the expression of fluorescent reporters.

## Background

The principle of epistasis has been an important tool in functional genomics and genetics research for more than a century [1,2]. According to this principle, genes may be defined as epistatic to one another when the phenotypic impact associated with a given mutation is altered by the presence of a second gene mutation. By measuring epistasis scores, which quantify departure from a given neutrality model marking the absence of epistasis (reviewed by [3]), it is possible to delineate genes functioning within common or parallel pathways and to infer regulatory hierarchies or functional complexes [4-10]. For example, aggravating interactions, which occur when the phenotypic impact of the double deletion is greater than predicted by neutrality, may result from the loss of compensatory pathways. Alternatively, alleviating interactions, which occur when the phenotypic impact is less than expected, may indicate that genes function within a common pathway or complex.

While epistasis reflects the structure of genetic networks in a given environment, the sign and strength of these interactions are expected to change in accordance to the substantial changes in physical interactions observed in response to external perturbations (see e.g. [11,12]). Such changes are anticipated to reflect the activation or inactivation of different pathways across environments. Indeed, it is has been well established that epistasis depends on both genetic and environmental contexts [8,9,13,14]. Interestingly, while the phenotypic impact of a changing environment is extensively analyzed in studies of gene-drug and drug-drug interactions (see e.g., [15-17]), the environmental modulation of epistasis between genes has received much less attention. Importantly, the analysis of fitness phenotypes may not enable a focus on pathways responding to specific environmental perturbations if the mutant strains involved have fitness defects in both the presence and absence of the perturbation [8].

To address this issue, we have developed a method from first principles to specifically identify pair-wise genetic interactions that change dynamically between environments. This analysis of gene-gene-environment interactions is similar to the generalization of epistasis in terms of three-dimensional genotopes [18]. We developed the method by explicitly incorporating environmental effects into the neutrality function used to identify epistatic relationships. It turns out that the derived neutrality function can be expressed in terms of sensitivity phenotypes. The method may thus be viewed not only as an identification scheme, but also as providing a formal basis for the sub-classification of fitness-based genetic interactions recently proposed by St. Onge *et al. *[8].

To explore the utility of sensitivity-based epistatic analysis, we examined two comprehensive *Saccharomyces cerevisiae *datasets describing the phenotypic impact of single and double gene-deletion in the presence and absence of the DNA-damaging agent methyl methanesulfonate (MMS). For the purpose of inferring transcriptional regulatory networks, we generated and analyzed 342 mutant strains carrying single- and double-deletions of 26 transcription factor (TF) genes. These TFs were selected due to the availability of comprehensive datasets describing the impact of MMS on their binding to downstream genes, as well as the genome-wide changes in MMS-induced differential gene expression following TF deletion [12]. As a preamble, we derive the classical multiplicative neutrality function and perform a conventional fitness-based epistatic analysis to identify genetic interactions in the both presence and absence of MMS. We also discuss in more detail why the results of a fitness-based epistatic analysis should not be used on its own to determine if a genetic interaction plays a role in a given cellular response. We then derive the sensitivity-based neutrality function by adopting the common assumptions that genetic and environmental perturbations can be treated equivalently [15-17], and that gene-environment interactions should remain invariant across genotypes in the absence of context-dependent epistasis.

Using the data obtained for single and double TF deletion mutants, we show that sensitivity-based epistatic analysis implicates a set of genetic interactions in the MMS-induced DNA damage response that is significantly different from that obtained using fitness phenotypes. Notably, only ~50% of the interactions identified using fitness phenotypes are also among those identified using sensitivity. A direct quantitative comparison of the two sets confirms that the sensitivity-based analysis specifically identifies interactions that change between environments. To explore this further, we compare sets of sensitivity- and fitness-based genetic interactions with datasets generated by Workman *et al *[12] describing MMS-induced differential gene expression and protein-DNA interactions in the presence of MMS. This comparison demonstrates that sensitivity-based epistatic analysis can improve the identification of environmental-dependant regulatory relationships within transcriptional regulatory networks.

To evaluate the utility of sensitivity-based epistatic analysis for the identification of functional relationships among DNA repair genes, we analyzed a dataset generated by St. Onge *et al *[8]. This dataset describes the phenotypic impact of MMS treatment on 349 single and double mutants carrying deletions of 26 genes conferring resistance to MMS. We demonstrate that hierarchical clustering of sensitivity-based epistasis signatures captures the composition and order of complexes and pathways with known roles in the DNA damage response. We also show that a sensitivity-based approach performs better than a fitness-based analysis for the identification of multi-component protein complexes with known functions in drug-induced DNA damage repair.

Taken together, our results suggest that sensitivity-based epistatic analysis may provide a useful tool to map how environmental perturbations modulate the architecture of genetic networks and reveal new insight into the regulatory networks and pathways mediating cellular responses to changing environments.

## Results and Discussion

### Fitness-based epistatic analysis

The identification of genetic interactions using fitness phenotypes is typically based on the expectation that the absence of epistasis is marked by the equality:

(1) W(X,Y)×W(wt)=W(X)×W(Y),

where *W*(*wt*), *W*(*X*), *W*(*Y*) and *W*(*X*, *Y*) are the fitness of the reference strain (wildtype, *wt*) and its single- and double-deletion derivatives, respectively. This relationship, which is attributed to Fisher [1], can be derived by comparing fitness defects caused by deleting gene *X *in the wildtype strain, defined by δ*W*(*X, wt*) = *W*(*X*)/*W*(*wt*), and a strain in which gene *Y *is also deleted, defined by δ*W*(*X*, *Y*) = *W*(*X*, *Y*)/*W*(*Y*). The equality in Eq. (1) is then obtained by assuming that the fitness defect caused by the deletion of *X *is independent of the presence or absence of gene *Y*, i.e., by setting δ*W*(*X, wt*) = δ*W*(*X*, *Y*). Defining fitness in terms of relative growth rates, Eq. (1) predicts that the growth rate *m*(*X*, *Y*) of the double mutant strain in the absence of epistasis is given by:

(2) $$m{\left(X,Y\right)}_{\exp}=\frac{m(X)\times m(Y)}{m(wt)},$$

where *m*(*wt*), *m*(*X*) and *m*(*Y*) are the growth rates of the wildtype and single mutant strains, respectively. The strength of an epistatic interaction can correspondingly be defined as the relative difference between the observed and expected double mutant growth phenotype:

(3) $$\varepsilon_{\text{fit}}=\frac{m(X,Y)-m{\left(X,Y\right)}_{\exp}}{m{\left(X,Y\right)}_{\exp}}=\frac{m(X,Y)\times m(wt)}{m(X)\times m(Y)}-1.$$

We refer to Eq. (3) as the fitness-based epistasis score (F-score) since relative growth rate fitness and growth rates can be used interchangeably.

**Figure 1:**
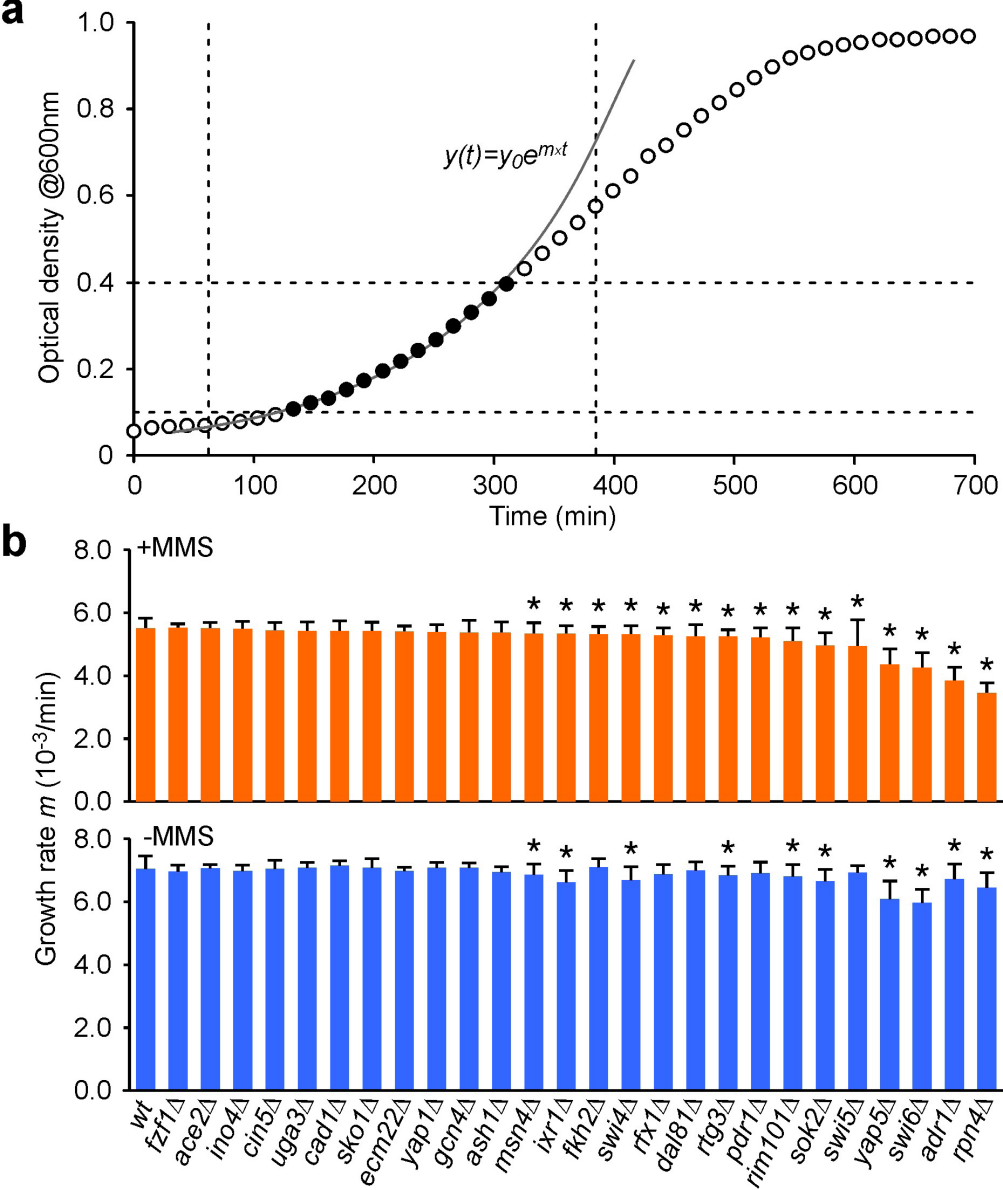
**Growth rate measurements**. (**a**) Representative optical density time course (open circles) illustrating the data used to estimate the logarithmic growth rate (filled circles). (**b**) Growth rates of the single TF deletion mutants in the presence and absence of MMS. Error bars indicate standard deviation. Asterisks indicate strains with altered growth rates compared to the wildtype (T-Test; *P *< 0.05).

To conduct a fitness-based epistatic analysis, we measured the growth rates of 342 single- and double-deletion TF mutants in the absence and presence of MMS (Figure 1a and Methods). Detailed results are provided in Additional File 1. Among the 26 single mutant strains, 15 had growth rates significantly different from that of the wildtype strain (Figure 1b, T-Test; *P *< 0.05). Eleven of 14 TF mutants identified as MMS sensitive in the study performed by Workman *et al. *[12] are also identified in our screen. The three mutants "missing" from our set (*ecm22Δ*, *gcn4Δ*, and *yap1Δ*) all have *P*-values just above threshold (*P *= 0.056, 0.055 and 0.080, respectively). Despite using conditions and methods that are significantly different, the overlap is comparable to that between the Workman study [12] and one by Begley *at al *[19] where 12 of 17 strains were identified in both studies using the same approach.

**Figure 2:**
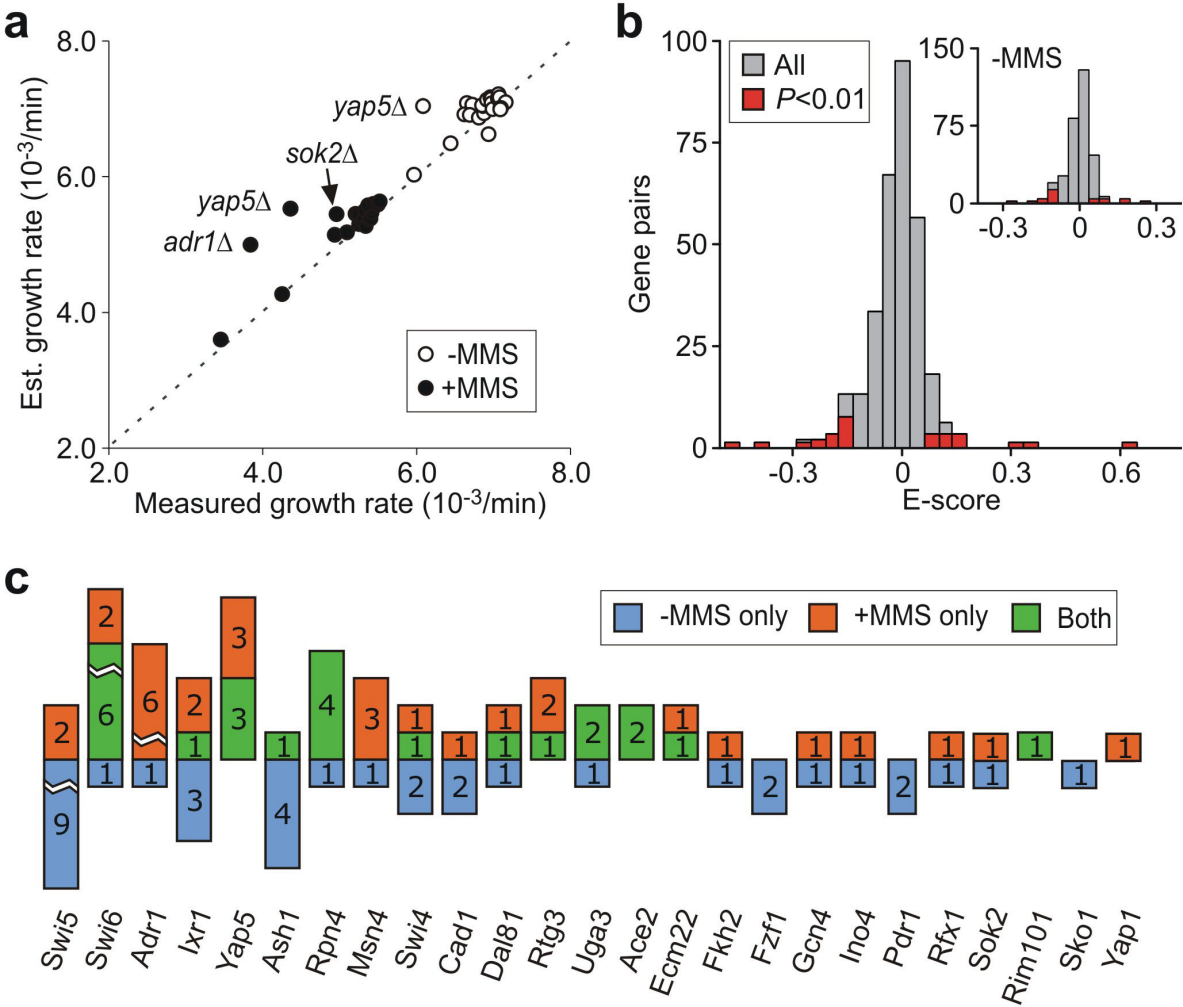
**Fitness-based epistatic analysis**. (**a**) Correlation between measured and estimated single mutant growth rates. (**b**) Histograms of fitness-based epistasis scores (F-Score) calculated for all TF-TF pairs (grey) and those associated with high confidence genetic interactions (red) in the presence of MMS. Insert displays the corresponding histograms in the absence of MMS. (**c**) The number of epistatic interactions identified for each TF, categorized according to the environment where the interaction was identified. 18 interactions are identified exclusively in absence of MMS, 15 are identified only in the presence of MMS and 12 are identified in both environments.

Fitness-based epistatic analysis can be performed using the measured single mutant growth rates directly [8], or by estimating the expected phenotypic outcome of double gene deletion from pooled fitness measurements [20]. Both approaches have their advantages and disadvantages. While the former is associated with uncertainty arising from alterations in growth phenotypes during the strain generation procedure [21], the latter requires a low frequency of growth defects and genetic interactions. Since the frequency of statistically significant growth defects is high within the TF single mutant library, we employ a variant of the pooling method in which growth rates of the single mutant strains is estimated from the median double mutant growth rate corrected for the phenotypic impact of the second deletion (see Methods). In most cases, the estimated single mutant growth rates obtained using this method is consistent with their directly measured values (Figure 2a). However, certain strains (*yap5*Δ, *sok2*Δ and *adr1*Δ) had deviations greater than 5%. This deviation could indicate a high number of epistatic interactions, or that a systematic bias was introduced during the generation of the double mutants. For example, the *yap5*Δ single mutant grew consistently slower than its double mutant progeny, suggesting that the mutant might carry a secondary mutation that is lost following mating. To mitigate the impact of such experimental uncertainties, we used estimated growth rates for the *yap5*Δ, *sok2*Δ and *adr1*Δ strains in our subsequent analyses.

The results of the fitness-based epistatic analysis are summarized in Figures 2b and 2c. Detailed results regarding F-scores obtained in both the presence and absence of MMS, as well as their associated *P *values, are provided in Additional File 2. Following strain generation, 316/325 of the possible double deletion strains were obtained for analysis. Figure 2b shows the histograms of F-scores for the corresponding TF-TF pairs in the presence and absence of MMS, as well as 45 interactions identified when the criteria *P *< 0.01 is used to reject the null hypothesis that epistasis is absent (see Methods). As expected, the F-score distributions are centred at zero in both environments (the median ε_fit _is 0.007 and 0.0012 in the absence and presence of MMS, respectively), and scores associated with identified interactions are located in the tails of these distributions. Of the 45 interactions identified, a significant fraction (27/45) is identified in the presence of MMS while the remaining interactions are identified only in its absence.

The association of genetic interactions with specific environmental conditions using F-scores does not necessarily support correct interpretations about their environmental dependency. For example, the identification of an interaction in both the presence and absence of MMS does not inherently indicate an MMS-independent relationship. While the interaction may be conserved across most environments, it could be of particular importance in a specific environment. For example, genetic interactions important for the maintenance of chromosome integrity in all environments may be critical for the repair of MMS-induced DNA damage. Conversely, it should not be concluded that an interaction is important for the MMS-induced response based on its identification exclusively in the presence of MMS. Several non-biological factors can contribute to a differential identification across different environments. For example, the true variance may by chance be over- or underestimated in one of the two environments. This may in turn cause the *P *value to be above its critical value in one environment and below it in the other. Within our dataset, we found that the variance among replicates is increased in the presence of MMS (data not shown), which inevitably introduce a bias towards identifying interactions in its absence. For these reasons, it is not possible to conclude if a given genetic interaction plays a role in pathways responding to specific environmental perturbations based solely on the measurement of fitness in the presence of the perturbation.

### Quantifying gene-environment interactions

To derive a neutrality function that incorporates environmental effects, it is noted that the phenotypic impact of changing the environment should be independent of a gene deletion when the mutated gene is not involved in the cellular response to this change. The principle of epistasis can thus be extended to gene-environment interactions when it is assumed that genetic and environmental perturbations can be modelled equivalently with respect to their impact on fitness, an assumption frequently employed in chemical biology (see e.g., [15-17]). To quantify the strength of gene-environment interactions analogously to that of genetic interactions, let the fitness defect caused by changing environment from *E*1 to *E*2 be given by δ*W*(*wt,ΔE*) = *m*(*wt*, *E*2)/*m*(*wt*, *E*1) in the presence of gene *X *and by δ*W*(*X,ΔE*) = *m*(*X*, *E*2)/*m*(*X*, *E*1) in its absence. When mutating gene *X *has no impact on the environmental response, i.e. δ*W*(*wt,ΔE*) = δ*W*(*X,ΔE*), the absence of a gene-environment interactions is marked by the equality:

(4) W(X,E2)×W(wt,E1)=W(X,E1)×W(wt,E2).

Equation (4) describes a neutrality function parallel to Eq. (1) in which a genetic perturbation has been substituted by an environmental perturbation to identify an interaction between a gene and the environmental condition rather than between genes. Using relative growth rate fitness, the expected growth rate of the mutant strain is in turn given by:

(5) $$m{\left(X,E2\right)}_{\exp}=\frac{m(X,E1)\times m(wt,E2)}{m(wt,E1)}.$$

Defining sensitivity as the ratio of growth rates in the two environments, *S *= *m*(*E*1)/*m*(*E*2), the relative difference between the observed and expected growth rate of the doubly perturbed strain can be written as:

(6) $$\varepsilon_{\text{env}}=\frac{m(X,E2)\times m(wt,E1)}{m(X,E1)\times m(wt,E2)}-1=\frac{S(wt)}{S(X)}-1.$$

We refer to Eq. (6) as the environmental sensitivity score (ES-score) since it quantifies the relative change in sensitivity to a new environment caused by a single genetic perturbation. Neutrality between gene *X *and the environmental change is inferred when deleting the gene has no impact on sensitivity, i.e., when ε_env_(*X*) = 0. Conversely, a non-zero ES-score implicates the gene in the cellular response to the environmental perturbation.

**Figure 3:**
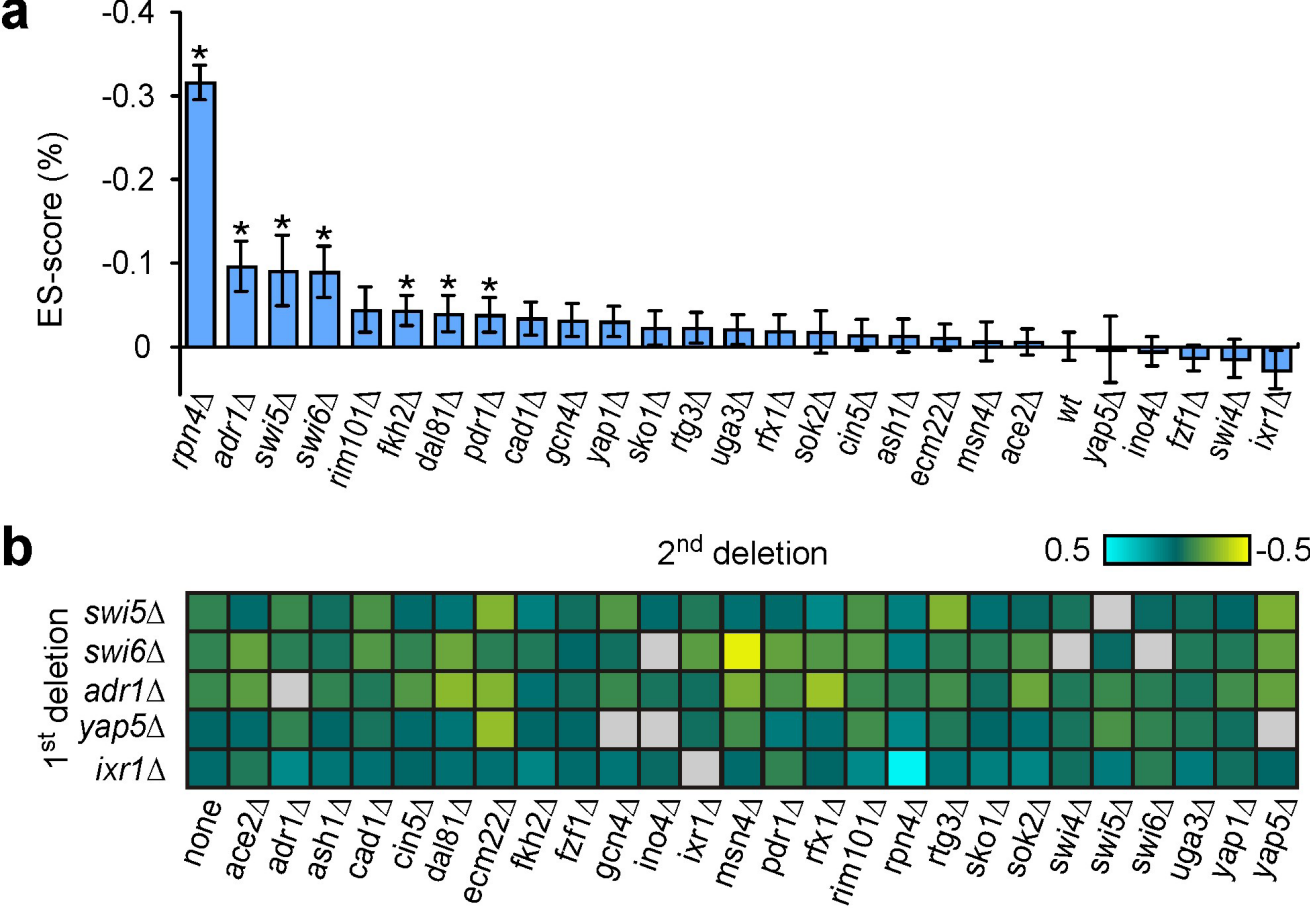
**Analysis of environmental sensitivity scores**. (**a**) Mean ES-scores for the wildtype (*wt*) and the 26 single deletion mutants. Asterisks indicate strains with statistically significant environmental sensitivity (*P *< 0.05). (**b**) Examples of variation in ES-scores for selected deletion mutants following the introduction of a second TF deletion. Grey squares indicate double mutants not assayed.

To identify which of our TFs are involved within the cellular response to MMS, we calculated ES-scores for the 26 single mutant strains (Figure 3a). Seven of these mutants have *P*-values indicating a significant interaction (*P *< 0.05), including *rpn4Δ*, which displayed the greatest effect (ε_env _= -0.31), and *adr1Δ*, *dal81Δ, fkh2Δ*, *swi5Δ*, *swi6Δ *and *pdr1Δ*, which displayed mild effects (ε_env _between -0.04 and -0.10). Noticeably, all displayed fitness defects in the presence of MMS (Figure 1). Conversely, not all strains associated with a fitness defect in the presence of MMS are accompanied by a high ES-score. Since the ratio of sensitivities in Eq. (6) may be expressed as a ratio of fitness values between the two environments, the relative impact of the mutation must be different across the two environments for the ES-score to assume a significant value. Consistent with this interpretation, with the exception of *rfx1Δ*, the eight mutants that display fitness defects in the presence of MMS but have low ES-scores also display fitness defects also in the absence of MMS (see Figure 1).

### Sensitivity-based epistatic analysis

Extending the definition of the ES-score in Eq. (6) to genetic backgrounds other than wildtype enables the identification of genetic interactions that change dynamically between environments. To demonstrate this, we note that the ES-score associated with deletion of gene *X*, in a strain that lacks gene *Y *is given by:

(7) $$\varepsilon_{\text{env}}=\frac{m(X,Y,E2)\times m(Y,E1)}{m(X,Y,E1)\times m(Y,E2)}-1=\frac{S(Y)}{S(X,Y)}-1.$$

In Figure 3b, we illustrate that the ES-scores associated with specific TF deletions can vary considerably in the presence of a second TF deletion. In the plot, we include only the TFs with a high number of fitness-based epistatic interactions to specifically highlight the variation of environmental sensitivity across different genetic backgrounds.

To derive a neutrality function that incorporates environmental effects, we impose the definition of epistasis by assuming that mutating gene *Y *should not affect the phenotypic impact of mutating gene *X *when the two genes act independently. Considering the impact on sensitivity following deletion of gene *X *as the phenotype preserved across different genotypes, it immediately follows from the equality ε_env_(*X*) = ε_env_(*X*, *Y*) that the absence of epistasis is marked by a sensitivity-based neutrality function where:

(8) S(X,Y)×S(wt)=S(X)×S(Y).

Equation (8) is a direct analogue of the fitness-based neutrality function in Eq. (1) and the strength of the interaction between genes *X *and *Y *can correspondingly be quantified by the sensitivity-based epistasis score (S-Score):

(9) $$\varepsilon_{\text{sen}}=\frac{S(X,Y)-S{\left(X,Y\right)}_{\exp}}{S{\left(X,Y\right)}_{\exp}}=\frac{S(X,Y)\times S(wt)}{S(X)\times S(Y)}-1.$$

where *S*(*X*, *Y*)_exp _is the sensitivity satisfying Eq. (8) expected under the null hypothesis that epistasis is absent.

**Figure 4:**
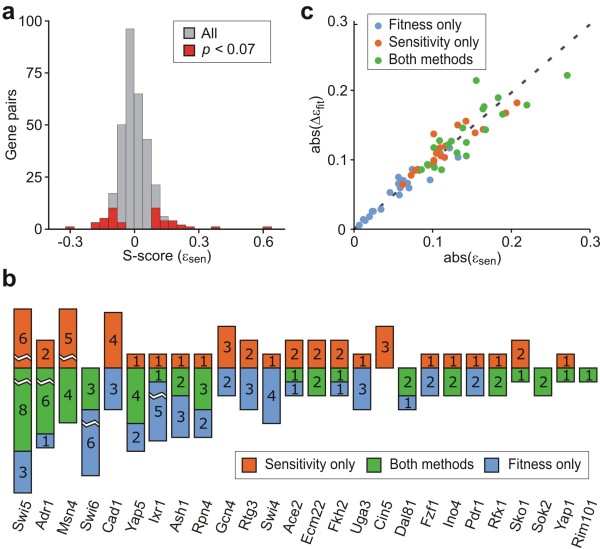
**Comparison of fitness- and sensitivity-based epistatic analysis**. (**a**) Histograms of sensitivity-based epistasis scores (S-Score) for all TF pairs (grey) and the 45 most likely epistatic interactions (red). (**b**) The number of epistatic interactions identified for each TF categorized according to the methodology by which the interaction was identified. (**c**) Correlation between the absolute S-score and the absolute difference in F-scores in the presence and absence of MMS.

To compare and contrast the fitness- and sensitivity-based approaches, we identified the 45 most likely epistatic interactions using F- and S-scores, respectively. The results of sensitivity-based analysis are summarized in Figure 4a, which displays the histograms of S-scores for all TF-TF pairs and the 45 interactions with the lowest *P *values. As in the fitness-based calculation, the S-score distribution is centred at zero (median ε_sen _= -0.007) and S-scores associated with high-confidence interactions are located in the extreme tails of this distribution. Of the 45 interactions, 37 have *P *values below 0.05, while the remaining eight have *P *values between 0.05 and 0.07. The additional interactions are included only to allow for a comparison of interaction sets of equal size. Interestingly, only half of the fitness-based epistatic interactions (24/45) are among those also identified using sensitivity phenotypes (Figure 4b). When a *P *value of 0.05 is used as the significance threshold, 16 of the 37 interactions are identified exclusively by the sensitivity-based method. Sensitivity-based epistatic analysis thus provides a perspective on TF-TF interactions in the DNA-damage response that is significantly different from that provided by fitness-based analysis.

To further explore the differences between the two methods, we plot in Figure 4c the correlation between the absolute value of ε_sen _and the absolute change in ε_fit _across the two environments. This plot demonstrates that the strength of F-scores associated with interactions not identified by sensitivity vary little between the two environments. One example is the interaction between the cell cycle regulators *SWI6 *and *ASH1*, which have a strongly alleviating interaction in both environments (ε_fit _= 0.25 and 0.33, respectively), but has a low S-score (ε_sen _= -0.06). In contrast to this, interactions identified solely by the sensitivity-based method involve an apparent change in the epistatic relationship between the two genes following MMS treatment. An example includes the interaction between the homologues, *ACE2 *and *SWI5*, which have well-documented overlapping functions in cell cycle regulation [22]. All interactions highlighted by the sensitivity-based method involve a marked change in F-scores between the two environments. For example, the four *SWI6 *interactions identified by both methods have F-scores that are high in one environment and low in the other. This includes the interaction between *SWI6 *and *MSN4 *interaction, which is weak in the absence of MMS (ε_fit _= -0.03) and strongly aggravating in its presence (ε_fit _= -0.33), resulting in a high S-score (ε_sen _= 0.6). Thus, sensitivity-based epistatic analysis allows for an assessment of the dynamic change in epistasis following an environmental perturbation. This may improve the identification of context-dependent regulatory relationships among genes, as well as the association of proteins to physical complexes and pathways involved in the response to environmental change.

### Inferring regulatory relationships

**Figure 5:**
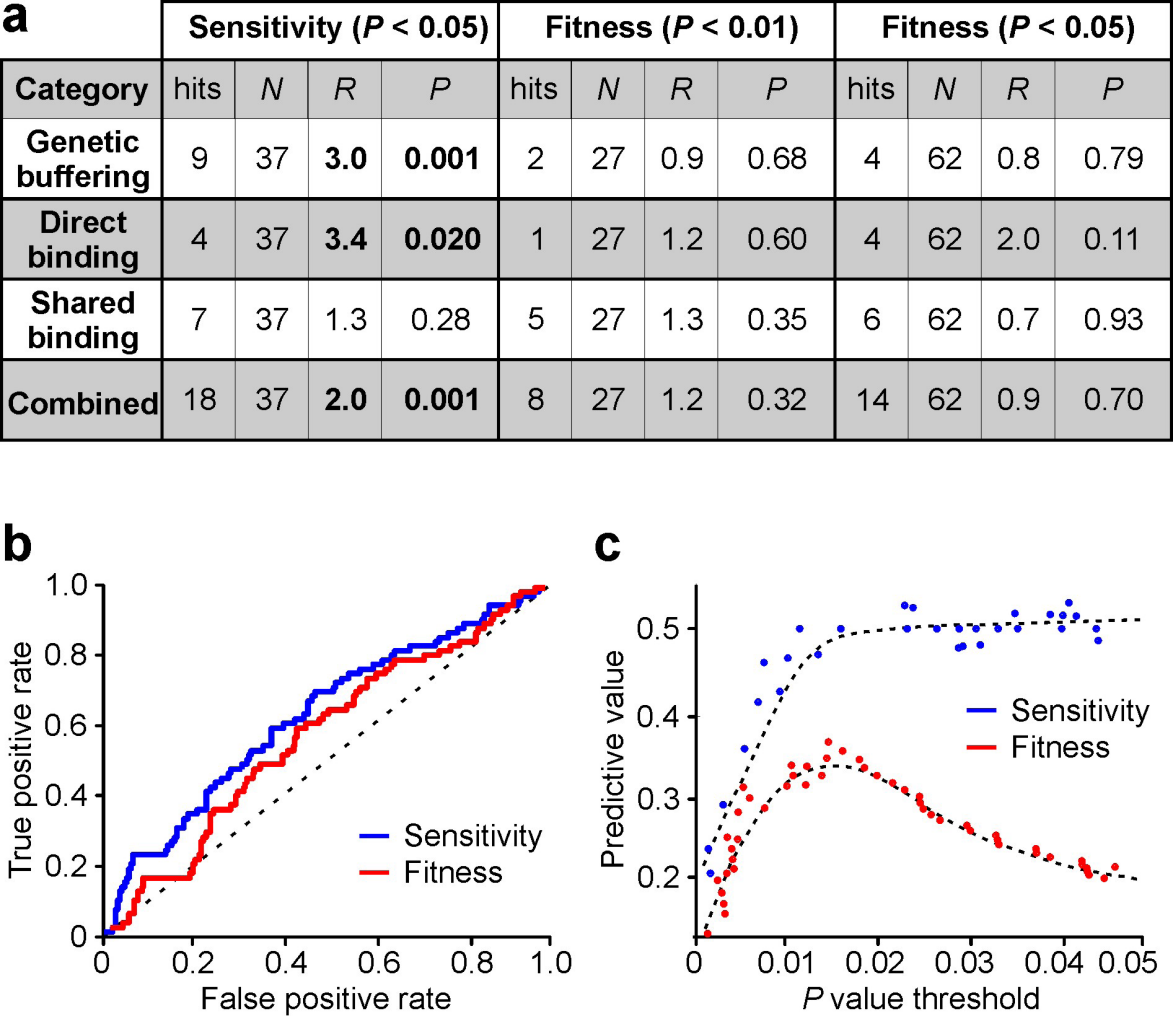
**Statistical comparison of genetic interaction sets**. **(a) **Genetic buffering and genome-wide TF-DNA binding data (Workman dataset) is taken for evidence of putative regulatory relationships among TFs. *R *gives the fold enrichment defined as a ratio of frequencies relative to a random model, and *P *the probability of observing a ratio of equal or greater value by chance. Boldface is used to indicate statistically significant enrichments (*P *< 0.05) within the genetic interaction sets relative to the frequency (hits/*N*) of interactions within the Workman data. Enrichment is analyzed in four categories: (1) Genetic buffering of one TF by another, (2) Direct binding of one TF to the gene encoding another, (3) TF pairs that bind to the same target gene(s), (4) interactions supported by any of the categories (1)-(3). The number of hits in the Workman dataset among the 316 pairs tested for each category are 26, 10, 46 and 77, respectively. **(b) **Comparison of true- and false-positive rates associated with each methodology. **(c) **The predictive value of the fitness- and sensitivity-based methods at different *P *value thresholds. The predictive value is defined as the fraction of correctly identified interactions.

To evaluate the utility of sensitivity-based epistatic analysis in identifying putative MMS-dependent regulatory relationships, we compared sets of interactions identified by fitness- and sensitivity-based epistatic analysis to datasets generated by Workman *et al *[12] describing the loss of MMS-induced differential gene expression following TF deletion, referred to as genetic buffering [12] or regulatory epistasis [23], as well as protein-DNA interactions in the presence and absence of MMS. To ensure a fair comparison, we used a set of sensitivity-based interactions identified with *P *< 0.05 and two sets of fitness-based interactions identified in the presence of MMS. The first containing 27 high-confidence (HC) interactions with *P *< 0.01, and the second containing 62 reduced-confidence (RC) interactions with *P *< 0.05. The results of this analysis are summarized in Figure 5a.

We first evaluated if the three sets of genetic interactions are enriched in direct genetic buffering whereby the deletion of one TF causes the loss of MMS-induced differential expression of another. Within the buffering dataset, there is evidence for genetic buffering interactions between 26 of the 316 TF pairs tested (*P *< 0.05). About one-third (9/26) of these direct buffering events are also identified by the sensitivity-based analysis corresponding to a significant 3.0 fold enrichment (*P *= 0.001, hypergeometric test). By contrast, the set of interactions identified using fitness phenotypes displays no significant enrichment over a random model (0.9 fold, *P *= 0.68 or 0.8 fold, *P *= 0.79 for the HC and RC sets, respectfully). In other words, if genetic buffering of one TF by another is viewed as evidence for a putative regulatory relationship, the sensitivity-based analysis clearly outperforms a fitness-based model in identifying such interactions.

To determine which genetic interactions are supported by physical interaction data, we analyzed the Workman protein-DNA interaction dataset focusing on genes that are differentially expressed following MMS treatment. We evaluated two scenarios where TF-DNA binding might manifest as a genetic interaction in the presence of MMS - the direct binding of one TF to another and the co-binding of two TFs to a common downstream gene. The analysis of direct binding to differentially expressed TF genes (identified using *P *< 0.05) provides evidence for putative regulatory relationships among 10 TF pairs. Four of these interactions are also identified in sensitivity-based set of interactions corresponding to a significant 3.4 fold enrichment (*P *= 0.02, hypergeometric test). By contrast, neither of the fitness-based sets displays enrichment.

To investigate the second scenario, we implemented a two-step analysis. First, for each genetic interaction, we performed a hypergeometric test by counting the number of differentially expressed genes bound by each TF and the number of genes bound by both. Here, the identification of differentially expressed genes uses a lower *P-*value (*P *< 0.01) to reduce the false positive rate. Within the set of interactions identified from sensitivity analysis, seven TF pairs display a significant enrichment in co-binding using a stringent cut-off of *P *< 0.01. To evaluate if this number of interactions is greater than expected from a random model, we counted the number of genes bound by any combination of TF pairs using the same criteria. This identified 46 TF pairs that are significantly enriched in co-binding among the 316 pairs tested. A hypergeometric test of these frequencies indicates no significant enrichment (1.3 fold, *P *= 0.28). Similar values are obtained for the fitness-based sets.

The most compelling evidence for the improved identification offered by sensitivity-based epistatic analysis is obtained by considering the totality of the Workman data. When direct buffering, direct binding and shared target binding are all considered evidence for a putative regulatory relationship among TFs, nearly 50% of the interactions identified by using sensitivity phenotypes are supported by at least one line of evidence (18/37 interactions, 2.0 fold enrichment, *P *= 0.001). By contrast, the sets of fitness-based interactions show no significant enrichment (Figure 5a). Some of the identified interactions are well established in the literature. One example is the interaction between *FKH2 *and *SWI5*, which, according to the *Saccharomyces *Genome Database, share a number of genetic interactions with genes involved in cell cycle progression. *FKH2 *is essential for the correct cell cycle periodicity of *SWI5 *transcription [24] and has been reported to prevent Swi5-specific activation of the cell cycle gene *CTS1 *[22]. Another notable example is the interaction between *SWI6 *and *RPN4*, which co-localize to several common genes and both buffers the mitochondrial DNA repair gene *DIN7 *[12]. The TFs also share 26 of 61 genetic interactions with genes that have MMS-specific phenotypes and documented roles spanning numerous DNA repair modules, including homologous recombination and post replication repair [25]. Existing genetic interaction data thus suggests that *SWI6 *and *RPN4 *are functionally linked in the MMS-response, in agreement with our observation of dynamic MMS-dependent genetic interaction between these genes.

To further compare the two methodologies, we calculated the true- and false-positive rates at varying *P *value thresholds when direct buffering, direct binding and shared target binding are all considered as evidence for a putative regulatory relationship. The results, which are displayed in Figure 5b, indicate that sensitivity-based analysis can improve the identification of regulatory relationships among TFs. Specifically, the sensitivity-based method identifies a higher number of true positives than the fitness-based method at any false-positive rate. This improvement becomes more evident when the predictive value, defined as the fraction of correctly identified interactions, is plotted for *P *values usually considered to imply statistical significance (Figure 5c). While the sensitivity-based method achieves a success rate of about 50% for *P *values between 0.01 and 0.05, the success rate associated with the fitness-based method at best is in the 25-35% range.

### Inferring functional complexes and pathways

To explore if sensitivity-based epistatic analysis can be used to identify functional complexes and pathways, we conducted hierarchical clustering of S-score profiles (see Methods). Clustering of S-scores calculated for the TF dataset did not yield meaningful results (data not shown) presumably due to the diverse and only partially overlapping roles of the different TFs in the MMS response. As an alternative, we analyzed a dataset generated by St. Onge *et al*. (2007) describing the fitness of 349 mutants carrying single- and double-deletions of 26 genes displaying fitness defects in MMS.

**Figure 6:**
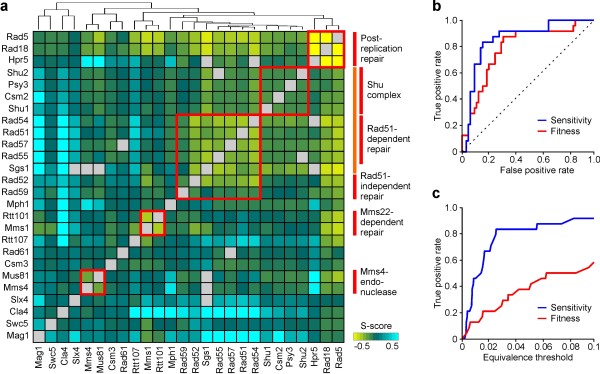
**Inference of functional modules and complexes relationships**. **(a) **Hierarchical clustering of S-score profiles. Red bars indicate genes in the Rad5 epistasis group, members of the Shu complex, the Rad51-dependent and independent branches of the Rad52 epistasis group, members of the Mms22-dependent pathway and the Mms4/Mus81 endonuclease complex. The orange bar highlights the clustering of genes in the Shu complex with the Rad51-dependent pathway and Sgs1 (see text for details). Grey squares indicate double mutants not assayed. **(b) **Comparison of true positive and false positive rates for fitness- and sensitivity-based identification obtained by varying the threshold (Δ*_thr_*) used to establish equivalence between single- and double-deletion phenotypes (see text). **(c) **Comparison of true positive rates for fitness- and sensitivity-based identification at different values of the equivalence threshold.

The hierarchical clustering of S-scores, displayed in Figure 6a, yields a grouping of the 26 genes that is consistent with known functional modules within the DNA damage response. These include members of the Rad6 epistatis group (*RAD5*, *RAD18 *and *HPR5*), which function within the post-replication repair (PRR) pathway [26,27], the Shu complex (*SHU1*, *PSY3*, *CSM2 *and *SHU2*) involved in promoting the formation of homologous recombination repair (HRR) intermediates [28], the Rad52 epistatis group (*RAD54*, *RAD51*, *RAD57*, *RAD55*, *RAD2 *and *RAD59*) involved in homologous recombination [29], as well as the Rtt101-Mms1 ubiquitin ligase [30] and the Mus81-Mms4 recombination factor [31]. As expected, the genes within these clusters have MMS-enhanced alleviating interactions with one another (negative S-score).

Interestingly, the sensitivity-based clustering places *SGS1 *within the Rad52 epistasis group in agreement with previous findings [32], but also appears to separate this group into two different components - one comprising *RAD54*, *RAD51*, *RAD57 *and *RAD55 *and the other comprising *RAD52 *and *RAD59*. The former group are members of the Rad51-dependent HRR pathway and function in parallel with members of the Shu complex to generate HRR intermediates processed by *SGS1 *[28]. The latter group is known to have additional functions in single stranded annealing not shared by the other members of the group [29]. It may therefore not be surprising that *RAD52 *and *RAD59 *cluster farther from the Shu genes and that SGS1 clusters together with *RAD54*, *RAD51*, *RAD57 *and *RAD55*. It is, however, interesting that hierarchical clustering of S-scores may be able to resolve how the different groups of genes act together within the MMS response. It is also interesting that the group comprising *RTT107*, which is grouped with Mms22-dependent repair in agreement with previous findings [33], *SLX4*, *CLA4*, and *MAG1 *display strong aggravating interactions with members of the group comprising *SGS1*, the Shu complex genes and the genes the in Rad51-dependent and independent HRR. This suggests that *RTT107*, *SLX4*, *CLA4 *and *MAG1 *may function in parallel to the main HRR pathway. In the case of *SLX4*, this is consistent with the finding that the Slx4 and Sgs1 are part of functionally redundant endonuclease complexes [34].

The clusters obtained using S-scores differ from those obtained by St. Onge *et al *[8] using fitness-based epistasis scores. Both analyzes correctly identify the functional relationships between *RAD5 *and *RAD18*, all members of the Shu complex, four members of the Rad52 epistasis group (*RAD52*, *RAD51*, *RAD55 *and *RAD57*), the linkages between *RTT107*, *RTT101 *and *MMS22*, as well as those between *MMS4 *and *MUS81*. However, the fitness-based clustering failed to reveal the functional relationship between *HPR5 *and members of the Rad6 epistasis group (*RAD5 *and *RAD18*), the involvement of *RAD54 *and *RAD59 *with other members of the Rad52 epistasis group, as well as the upstream role of Sgs1 in processing HRR intermediates generated by Shu complex and the Rad51-dependent HRR pathway. Notably, while analysis of fitness phenotypes performed by St.Onge *et al *identified an alleviating interaction between *HPR5 *and both *RAD5 *and *RAD18*, the use of sensitivity-based clustering appears to better capture the interplay of *HPR5*, with both the Rad6 pathway (see Figure 6a), consistent with the observation of direct physical interactions between *HPR5 *and both *RAD5 *and *RAD18 *[27].

To further compare the two methods, we evaluated their ability to correctly recover interactions among genes encoding multi-component protein complexes. Within a positively regulated pathway where *X *acts upstream of *Y*, deleting the upstream gene is expected to mask the phenotypic effect of deleting the downstream gene [4]. This phenotypic masking can be detected if *W*_XY _= *W*_X _or *S*_XY _= *S*_X_. In terms of epistasis scores, this corresponds to ε_fit _= *W*_wt_/*W*_Y_-1 when fitness phenotypes are used, and to ε_sen _= ε_env_(*Y*) = *S*_wt_/*S*_Y_-1 when sensitivity phenotypes are used. For genes encoding different components of a physical complex, it is further expected that ε_fit _= *W*_wt_/*W*_X_-1 and ε_sen _= *S*_wt_/*S*_X_-1, corresponding to co-equivalence among mutant phenotypes. Figure 6b compares the fitness- and sensitivity-based methods in recovering phenotypic masking among protein complex genes. We focussed on three putative multi-component protein complexes involving members of the Rad6 epistasis group (*RAD5*/*RAD18*/*HPR5*), the Shu complex (*SHU1*, *PSY3*, *CSM2 *and *SHU2*) and 3 members of the Rad51 HRR pathway (*RAD51*/*RAD57*/*RAD55*). The genes within each complex are annotated as interacting physically with one another according to the BioGRID database [35] and define a set of 24 directional interactions displaying phenotypic masking; two for each of the 12 gene pairs. Full data is provided in Additional File 3. We defined phenotypic masking as an alleviating interaction (*P *< 0.05) where the difference between respective single- and double- deletion mutants for fitness (Δ*_thr x _*= |ε_fit _-*W*_wt_/*W*_Y_+1| and Δ*_thr y _*= |ε_fit _-*W*_wt_/*W*_X_+1|) or sensitivity-based (Δ*_thr x _*= |ε_sen_-ε_env_(*Y*)| and Δ*_thr y _*= |ε_sen_-ε_env_(*X*)|) measurements are below a certain threshold (Δ*_thr_*). By applying this approach to test for phenotypic masking between all 636 directional interactions, the sensitivity-based identification outperforms that based on fitness phenotypes (Figure 6b). This is more clearly demonstrated in Figure 6c, which shows the fraction of masking relationship recovered when Δ*_thr _*is less than 10%. Indeed, for Δ*_thr _*= 0.1, the sensitivity-based approach recovers 92% (22/24) of the predicted interactions, including those between Rad5 and Rad18, all the members of the Shu complex, as well as the putative complex involving Rad51, Rad55 and Rad57. By contrast, the fitness-based approach recovers only the interactions among members of the Shu complex, which accounts for less than 60% of the predicted relationships.

## Conclusion

We have presented a method that extends conventional fitness-based epistatic analysis to specifically identify genetic interactions that are dynamically modulated in response to an environmental perturbation. The identification of such interactions may provide several advantages by allowing a focus on pathways responding specifically to a given environmental perturbation [8]. Noticeably, within the TF dataset analyzed, as few as ~50% of the interactions identified using fitness phenotypes are also identified using sensitivity. These interactions represent linkages among transcriptional regulators that change in a response-specific manner. Thus, combining the two approaches may enable the segregation of genetic interactions within pathways involved in specific cellular responses, and interactions associated with core processes preserved across environments. This conclusion is supported by the analysis of genome-wide profiling of MMS-induced changes in transcription and protein-DNA interaction data. This analysis demonstrates a clear enrichment in putative regulatory relationships among TF pairs identified by sensitivity-based epistatic analysis, a result not provided by the analysis of fitness phenotypes. Moreover, our analysis of epistasis within known DNA damage repair pathways confirms that quantifying the environmental dependency of genetic interactions can be used to associate genes with different functional groups, physical complexes and pathways. By applying this principle across a larger dataset encompassing additional environmental conditions, we anticipate that this methodology could aid in deciphering the dynamics of gene networks.

Integrating physical and phenotypic data into comprehensive and accurate models of regulatory networks and pathways remains a major challenge in systems biology [36]. The mapping of biomolecular interactions and transcriptional profiling provide fundamental insight into the substantial remodelling of gene regulatory networks that take place following environmental perturbations. However, it is not always clear if and how observed changes in the physical interaction network manifest at the physiological level. This can be clarified using the phenotypic information provided by sensitivity-based epistatic analysis since the dynamically modulated interactions identified by this method are likely to reflect the remodelling of network architecture in response to environmental cues. As such, the method may have important applications in the inference and analysis of biological networks.

## Methods

### Strains

Double gene deletion mutants were generated as described [37]. To construct a single-deletion "starter" strain library, the TF-encoding open reading frames were deleted using a PCR-based gene replacement strategy conferring uracil prototrophy or kanamycin resistance. Kanamycin-resistant single-deletion mutants derived from strain BY4741 (MATa *his3*Δ *leu2*Δ *met15*Δ *ura3*Δ) were obtained from Open BioSystems. Uracil prototrophic strains were derived from strain Y7092 (MATα can1Δ::STE2pr-LEU2 *lyp1*Δ *ura3*Δ0 *leu2*Δ0 *his3*Δ1 *met15*Δ0, kind gift of Dr. Kristin Baetz).

### Growth Assays

Glycerol stocks maintained at -80°C were thawed at 4°C and 20 μl used to inoculate 380 μl YPD media, containing 10 g/l yeast extract (Wisent), 20 g/l of Bactopeptone (Fisher), 20 g/l of dextrose (Fisher) and 0.042 g/l adenine (Sigma), followed by incubation overnight at 30°C under continuous shaking (250 rpm). 20 μl aliquots were subsequently diluted with 280 μl YPD and the optical density at 600 nm (OD) measured using a PerkinElmer Victor3 V 1420 Multilabel Counter following incubation at 30°C for 1.5 hours. The OD was then adjusted to ~0.16 by dilution with fresh YPD, and 35 μl added to 35 μl YPD or 35 μl YPD supplemented with 0.015% MMS (Sigma) in a 384 well plate. Each well was overlaid with a 6 μl layer of light mineral oil (Sigma) to minimize evaporation. Growth curves were estimated by measuring OD at ~15 minute intervals for 10 hours at 30°C in no less than 19 and 4 replicates for single- and double-deletion strains, respectively. A custom Matlab script was used to calculate growth rates from OD values in the range from 0.1 to 0.4, and obtained between 60 and 360 minutes after inoculation by fitting to an exponential growth model. Following manual inspection, growth rate estimates were computed based no less than 10 (-MMS) or 12 (+MMS) data points. A decreased OD window was used in a few cases to allow for the analysis of strains with slow initial growth. Single mutant growth rate were estimated from double mutant data using the following procedure. For each TF, a set of growth rates μ(*X*) = μ*_1_*(*X*)... μ*_N_*(*X*) was calculated from Eq. (1) under the hypothesis that epistasis with each of the other TFs is absent, i.e., μ*_i_*(*X*) = *m*(*X*, *Y_i_*)×*m*(*wt*)/*m*(*Y_i_*) where *Y_i _*refers to the second TF deleted. The single mutant growth rate is then estimated by the median of μ(*X*).

### Statistical analysis

Statistical significance was assessed using parametric bootstrapping. Simulated data, consisting of random numbers drawn from distributions with the same mean and variance as the experimental data was used to estimate the probability of observing an epistasis score as extreme, or more extreme than the observed epistasis score by chance under the null hypothesis that epistasis is absent. The null hypothesis was imposed on the simulated data by drawing the appropriate double mutant growth rate from a distribution with a mean (m_0_) given by the growth rate expected in the absence of epistasis and a variance given by m_0_^2^×(cv_1_^2 ^+ cv_2_^2^) where cv_1 _and cv_2 _are the coefficients of variation associated with the measured double mutant growth rate and the median coefficients of variation of all double mutant growth rates, respectively. P-values for each epistasis score was computed based on 300000 trials.

The assignment of fitness-based epistasis to specific environments (*E1*, -MMS; *E2*, +MMS) was based on the *P *values in the two environments. Interactions were associated with the absence of MMS if *P*_-MMS _< 0.01 and *P*_+MMS _> 0.01, to the presence of MMS if *P*_-MMS _> 0.01 and *P*_+MMS _< 0.01, and to both environments if *P*_-MMS _< 0.01 and *P*_+MMS _< 0.01. The *P *values associated with protein-DNA interactions and loss of differential expression in TF deletion strains were provided by Dr. Trey Ideker and analyzed as described [12].

### Hierarchical clustering

The analysis was performed in the *R *programming language using the pvclust package with default parameters (correlation-based measure of dissimilarities between objects, and agglomeration based on averages) [38].

## Abbreviations

TF: transcription factor; MMS: methyl methansulfonate; F-score: fitness-based epistasis score; ES-score: environmental sensitivity score; S-Score: sensitivity-based epistasis score; HC: high-confidence fitness-based epistatic interactions; RC: reduced-confidence fitness-based epistatic interactions; YPD: Yeast Peptone Dextrose.

## Authors' contributions

CB and MK designed the research. CB and LT generated and validated the mutant strains. CB and MK performed data analysis. CB and MK wrote the manuscript. All authors have read and approved the manuscript.

## Supplementary Material

Additional file 1 **Analysis results for single TF mutant data**. Tab-delimited text file containing information on single mutant growth phenotypes in the presence and absence of MMS. Click here for file

Additional file 2 **Epistatic analysis results for TF mutants**. Tab-delimited text file containing calculated F- and S-scored as well as associated P-values computed for double mutant strains. Click here for file

Additional file 3 **Results for phenotypic masking**. Tab-delimited text file containing information on measured and predicted F- and S-scores used to identify masking interactions. Click here for file
